# Supplementary material for: Learning curve and surgical outcome of robotic assisted colorectal surgery with ERAS program
Source: Sci Rep. 2022 Nov 29;12:20566. doi: 10.1038/s41598-022-24665-w (PMC9709162; doi:10.1038/s41598-022-24665-w)
Supplement: Supplementary file 1 — Supplementary Information 1. [file 41598_2022_24665_MOESM1_ESM.zip › LSC Raw data-Ñ[▒K/097/O_ñw╝╨░O▒Kñσ.pdf]

正式摘要(電子簽章：000000201329593)

W52 / W52

臺 中 榮 民 總 醫 院

Operation Note

[redacted]

- 手術日期：2020/10/15
- 手術醫師：VS 蔣鋒帆，R 陳琦夫，R 楊捷儒
- Anesthesia：GE

Diagnosis

- Pre-operative diagnosis : 1. Squamous cell carcinoma in situ arising in condyloma acuminatum s/p total anoplasty on 2015/01/30 ; s/p repeated resection and CCRT under our CRS and C/T service ; R/O tumor recurrence s/p resection on 2020/05/28
- Post-operative diagnosis : 1. Squamous cell carcinoma in situ arising in condyloma acuminatum s/p total anoplasty on 2015/01/30 ; s/p repeated resection and CCRT under our CRS and C/T service ; R/O tumor recurrence s/p resection on 2020/05/28, s/p laparoscopic APR on 20201015

Surgical Indication

- For curative resection

Operation

- Abdominoperineal resection for neogrowth of anorectum (APR) Laparoscopy

Finding

- 1. A ulcerative tumor about 3 X 6cm in size at low rectum, more than 1cm FAV.
  2. LN : (-)
  3. Liver metastasis: (-)
  4. Ascites : (-)
  5. Cut margin: proximal 20 cm, distal more than 1 cm.
  6. Laparoscopic abdominal-perineal resection with Endo GIA support
  7. High ligation (+) ; The ligation site was at IMA root area
  8. Take down of splenic flexure: Yes
  9. 12 Fr. penrose drain (The drain was divided equally into 2 pieces, serve as two drain) was placed at perineum space

Procedure

- 1. General endotracheal anesthesia was induced and then the patient was placed in modified Trendelenburg lithotomy position. The abdomen was prepped and draped in standard sterile fashion.
  2. A small vertical incision was made near the umbilicus and a 12mm trocar was inserted into the peritoneal cavity by open method. Peumoperitoneum was established with CO2 inflation.
  3. There was no severe adhesion, and another another 12mm trocar was inserted over RLQ of abdomen smoothly ; Followed by other 5mm trocars (X2) over RUQ and LLQ of abdomen.
  4. The operative findings were mentioned as above.
  5. The inferior mesentery artery was identified, ligated near its origin from the aorta, above the left colic artery with endoclips, and transected, so was inferior mesentery vein.
  6. Dissection was done above the Gerota's fascia and toward the peritoneal attachments.
  7. The colon was mobilized from retroperitoneum overlying the sacral promontory and into the pelvis. The incision was extended laterally and medially across the peritoneal reflection.
  8. The mesorectum was then mobilized using sharp dissection below the level of pelvic floor muscle, near dentate line. Then distal descending colon and rectum were mobilized.
  9. An endoscopic stapler was then used to divide the sigmoid and then laparoscope was removed.
  10. We further performed perineal phase of dissection.

- 11. An elliptical incision was made encompassing the anus and extending up to posterior wall of prostate.
  12. Dissection then progressed through through subcutaneous tissues circumferentially. The pelvic cavity was entered posterior to the anus in the midline.
  13. The levators were then divided with electrocautery beginning posteriorly and progressing anteriorly. The specimen was delivered through this posterior incision into the perineal field.
  14. The remaining anterior attachments were then severed and the specimen was removed. Hemostasis was achieved in the pelvis and perineal wound.
  15. Surgiwrip was applied over the perineal area ; Then two 12 Fr. penrose drains (It was equally divided from 1 drain) were placed at perineal and pelvic space via the incision wound.
  16. The fascia of the two 12 mm trocar wound was closed with 1-0 Vicry, and the skin was sutured with 3-O nylon, all covered with Menpor.
  17. The colostomy was then matured with multiple interrupted suture of 4-0 Monocryl placed in such a way as to tack the full thickness of the edge of the colon to a subcuticular layer of the skin.
  18. The patient tolerated the procedure well and was brought to the POR in stable condition.
- Estimated blood loss: 250 c.c
- 器械數量: 正確
- 紗布數量: 正確

主任 2062F蔣鋒帆 / 醫師 2310K陳璿夫

正式摘要(電子簽章：000000201329593)

W52 / W52

doctorRank:B
